# Pubosymphyseal Urinary Fistula Following Radiation Therapy of Bladder Sarcomatoid Tumor

**DOI:** 10.7759/cureus.46261

**Published:** 2023-09-30

**Authors:** Nathan Burriss, Abdul Rahman Abualruz

**Affiliations:** 1 Radiology, Medical College of Georgia at Augusta University, Augusta, USA

**Keywords:** urinary bladder ca, urosymphyseal fistula, pelvic radiation, symphysis pubis osteomyelitis, urinary fistula

## Abstract

Pubosymphyseal urinary fistula (PUF) is a rare condition that involves an abnormal connection between the urinary bladder and the pubic bone. It can occur after trauma, radiation therapy, or surgery to the pelvis. It is also reported with chronic indwelling Foley catheter use. In this case report, we present a 56-year-old male who developed a fistula complicated by osteomyelitis pubis following external beam radiation for a urinary bladder sarcomatoid tumor.

Patients at high risk of PUF may present with urinary leakage, pelvic pain, and infection, making diagnosis challenging. The condition can lead to chronic pelvic pain and long-term opioid use if left untreated. Diagnosis is typically made through imaging studies (CT scan or MRI) and confirmed with cystoscopy. Treatment usually involves urinary diversion/surgical repair of the fistula and management of any associated infection or complications. The prognosis is generally good if the condition is diagnosed and treated promptly.

While it is rare, it can have significant consequences that require prompt diagnosis and treatment.

## Introduction

Pubosymphyseal urinary fistula (PUF) is a rare complication reported more commonly following surgery or radiation treatment for pelvic malignancies [1]. PUF can be challenging to diagnose and can cause significant pelvic pain leading to chronic opioid use, recurrent urinary infections, osteomyelitis, and urosepsis [2]. Radiotherapy is a common and effective treatment for a range of pelvic tumors, including advanced bladder tumors [3]. However, it can also have adverse effects on pelvic organs, such as radiation cystitis in the urinary system, osteonecrosis, and pubis osteitis in the bones. Early recognition, prompt intervention, and coordinated treatment can help manage the patient's symptoms and improve their quality of life.

## Case presentation

A 56-year-old male with a medical history of type II diabetes complicated by peripheral neuropathy and toe amputation, hypertension, hyperlipidemia, chronic smoking, and benign prostatic hyperplasia (BPH) was under surveillance for microscopic hematuria. Urine cytology revealed malignant cells, and CT urography confirmed the presence of a large bladder mass with metastases to the pelvic sidewall lymph nodes. The patient underwent transurethral resection of the bladder tumor (TURBT), which revealed sarcomatoid bladder cancer.

**Figure 1:**
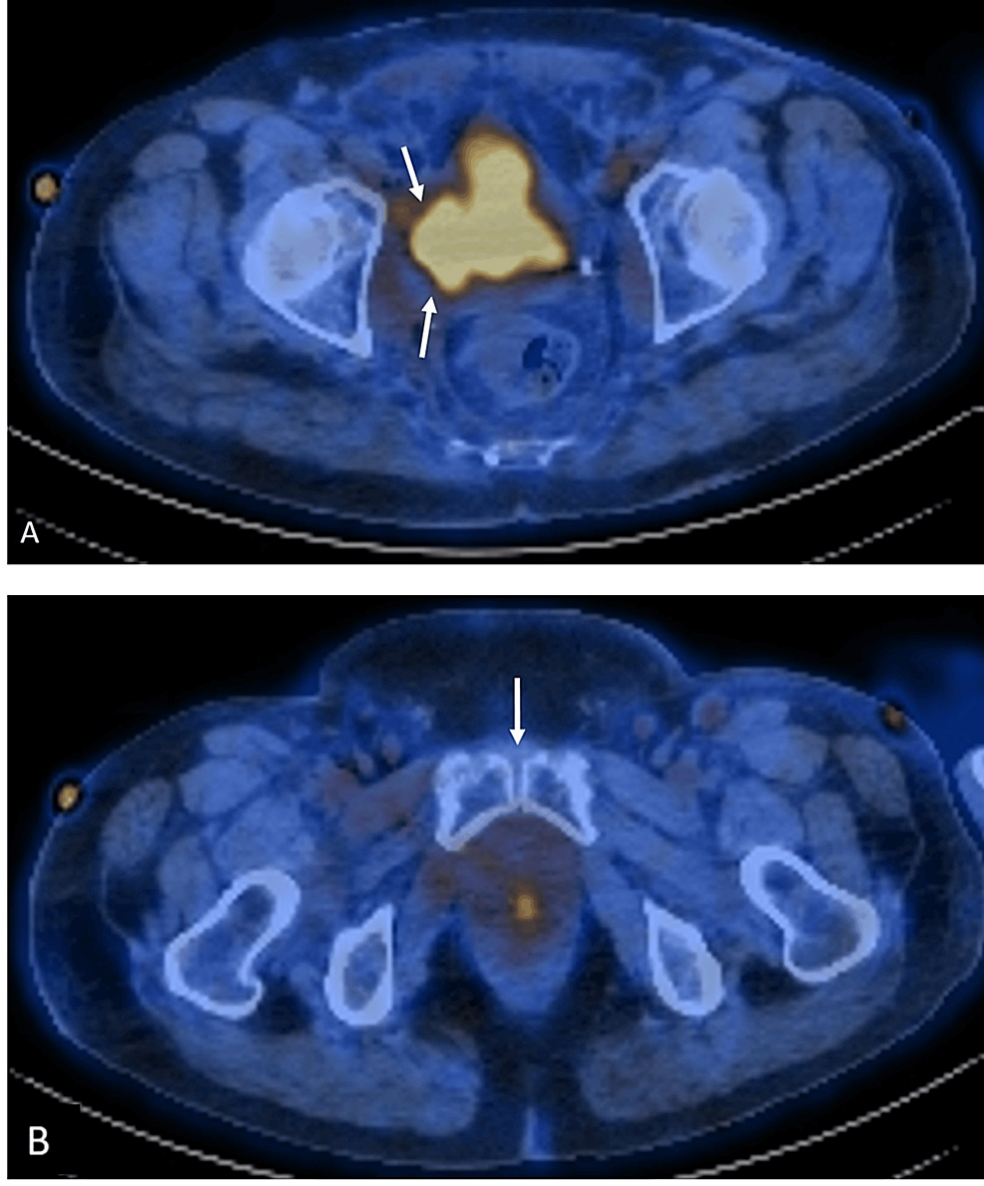
PET-CT images through the pelvis in the axial plane (A) PET/CT images demonstrate radiotracer accumulation in the urinary bladder with the extension of radiotracer into the right pelvic side wall (white arrows), suggestive of an underlying bladder wall defect. (B) Notice the lack of tracer extension into the symphysis pubis (white arrow). PET-CT: Positron emission tomography-computed tomography.

After TURBT, the patient received neoadjuvant chemotherapy with gemcitabine-cisplatin and fractional beam radiation to the bladder, with a total planned dose of 55 Gy. Unfortunately, treatment had to be halted due to renal failure and candida septicemia. The patient subsequently received nivolumab infusions and additional fractional external beam radiation, with a total dose of 40 Gy. Over two years following initial therapy, surveillance imaging showed stable disease until a positron emission tomography-computed tomography (PET-CT) scan revealed enlarging right pelvic sidewall nodal disease, for which external beam radiation to the right pelvic sidewall was initiated. Follow-up PET/CT demonstrated an excreted radiotracer in the urinary bladder, which extends into the right pelvic side wall (Figure 1), suggesting lateral bladder wall necrosis as an early sign of fistula development.

**Figure 2:**
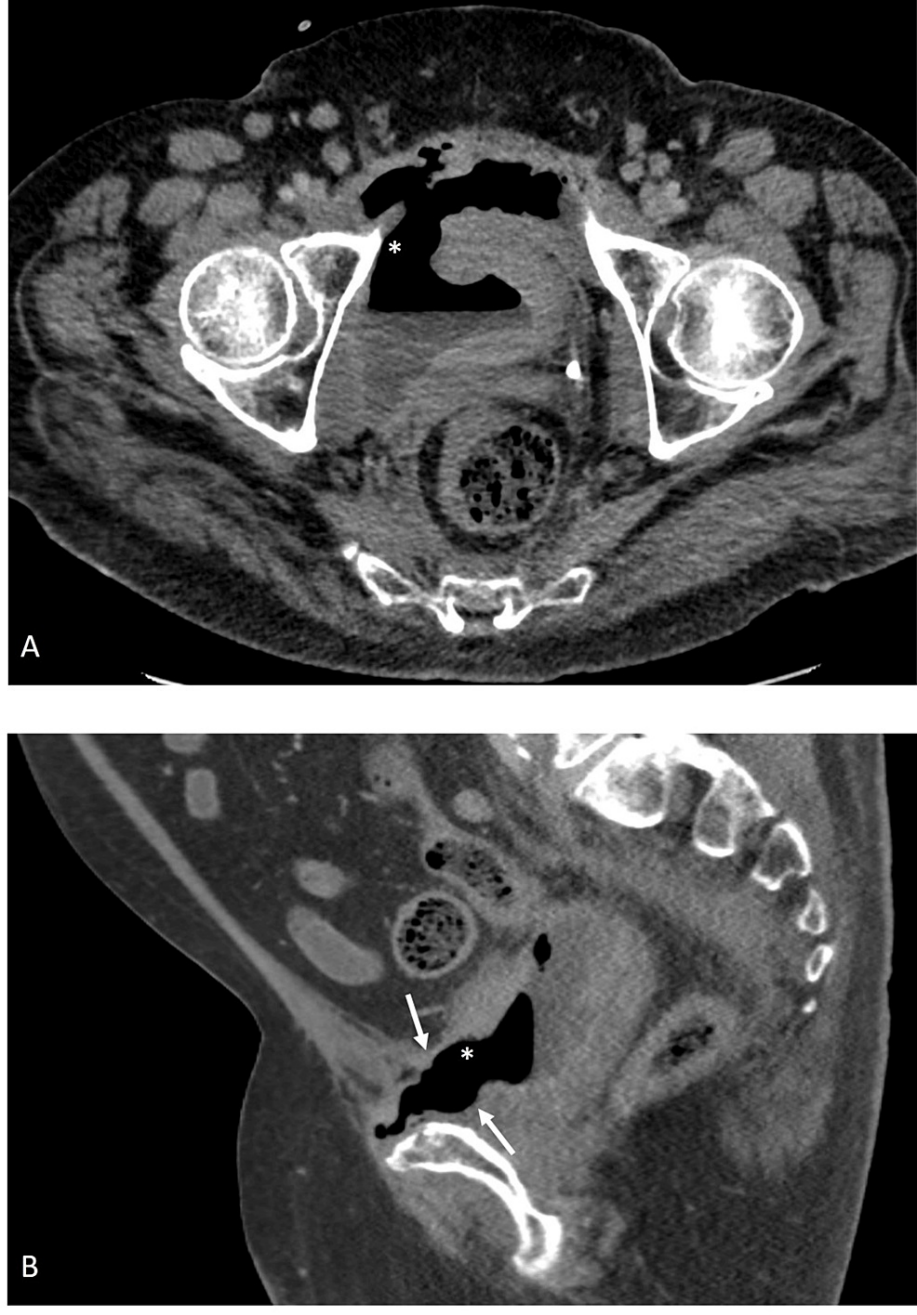
Contrast-enhanced CT images through the pelvis in axial (A) and sagittal (B) planes Contrast-enhanced CT images of the pelvis demonstrate direct fistulous communication (asterisk) between the urinary bladder lumen and the pubic symphysis (white arrows).

**Figure 3:**
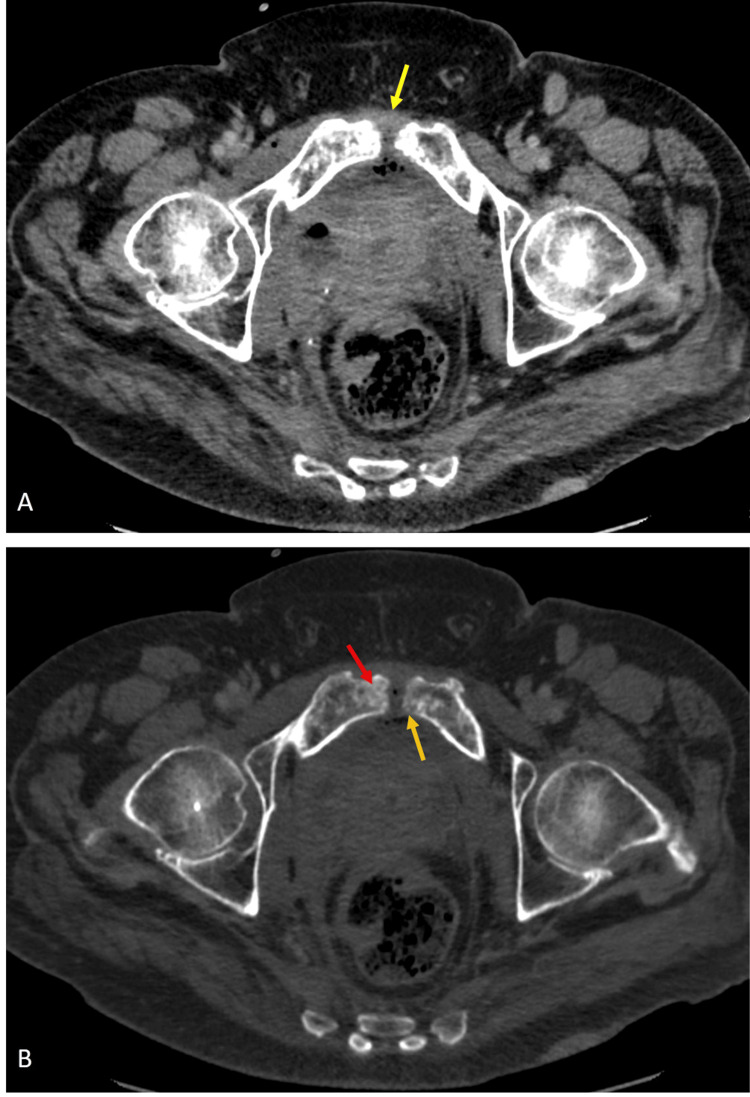
Contrast-enhanced axial CT images through the pelvis in soft tissue (A) and bone (B) windows (A) Contrast-enhanced axial CT images of the pelvis show a small volume of fluid and air locules at the symphysis pubis (yellow arrow), (B) in addition to bony erosions/destruction of the pubic body consistent with osteomyelitis (red arrow).

However, after the ninth fraction, the patient developed pelvic pain and sought emergency care. A CT abdomen and pelvis revealed abnormal communication between the urinary bladder and symphysis pubis (Figure 2) complicated by osteomyelitis pubis (Figure 3). As a result, radiation therapy was terminated, and the patient was started on antibiotics and urinary diversion to facilitate fistula healing. Following five months of nonsurgical management, the patient reported symptom resolution with no residual pelvic pain.

## Discussion

PUF is a rare and often overlooked complication that was initially reported in the literature following radical prostatectomy [4]. Later, it was identified as a complication of radiation therapy for prostate cancer and as a sequela of endoscopic procedures for radiation-induced posterior urethral strictures [2,4-6]. Recently, a case series published cases of women who developed PUF after radiation therapy for anal and endometrial cancer, along with chronic indwelling Foley usage [7]. Our case report highlights the growing recognition of this condition in patients receiving pelvic radiation for malignancies other than the prostate gland.

Sarcomatoid bladder cancer is an infrequent form of urothelial carcinoma, with only a few documented cases in the literature [8]. PUF-complicating urinary bladder tumors are exceedingly rare, with only four cases reported in one case series [9]. Our patient developed a PUF after external pelvic radiation for tumor recurrence, potentially due to the combined effect of prior treatments and weakened bladder wall.

Patients with PUF can present with pain in the pelvic area, urinary leakage, and infection. Diagnosis is typically made through imaging studies (CT scan or MRI) and confirmed with cystoscopy. Imaging with pelvic MRI or CT is essential in identifying the fistulous communication between the urinary bladder and symphysis pubis [6,10]. MRI is superior to CT for the detection of bone marrow edema in early osteomyelitis [10], which informs management decisions.

Treatment usually involves both nonsurgical and surgical options [1,6]. Nonsurgical management includes urinary diversion, antibiotics, and cystoscopic intervention. Urinary diversion can be achieved through suprapubic catheterization, urethral catheterization, or nephrostomy tube placement. Surgical interventions have been shown to markedly improve symptoms in most patients [1,6]. Surgical options involve fistula closure or reconstruction after debridement of necrotic/infected tissues, which showed high efficacy in chronic pelvic pain control in patients affected by PUF [6]. In our case, the patient received antibiotic therapy for osteomyelitis and urinary diversion with a complete resolution of symptoms five months after treatment.

The prognosis is generally good if PUF is diagnosed and treated promptly. However, it is dependent on several factors, including the underlying etiology, the extent of the injury, and the patient's overall health.

## Conclusions

PUF is a rare yet serious complication of pelvic surgery or pelvic malignancy treatment. Early PUF identification and treatment significantly enhance patients' quality of life. By increasing awareness of this condition, clinicians can expedite diagnoses, provide suitable therapy, and reduce patient morbidity.
